# Interleukin 10 gene polymorphisms and frailty syndrome in elderly Mexican people: (Sadem study)

**DOI:** 10.1002/mgg3.918

**Published:** 2019-08-06

**Authors:** Teresa Juárez‐Cedillo, Gilberto Vargas‐Alarcón, Nancy Martínez‐Rodríguez, Enrique Juárez‐Cedillo, José Manuel Fragoso, Jorge Escobedo‐de‐la‐Peña

**Affiliations:** ^1^ Comisionada en la Unidad de investigación en Epidemiologia Clínica, Hospital Regional No. 1, Dr. Carlos McGregor Sánchez Navarro, Instituto Mexicano del Seguro Social Mexico City Mexico; ^2^ Departamento de Biología Molecular. Instituto Nacional de Cardiología Ignacio Chávez, (SSA) Mexico City Mexico; ^3^ Community Health Research. Department Hospital Infantil de Mexico Federico Gomez; (SSA) Mexico City Mexico; ^4^ Unidad de investigación en Epidemiologia Clínica Hospital Regional No. 1, Dr. Carlos McGregor Sánchez Navarro, Instituto Mexicano del Seguro Social México City México

**Keywords:** frailty, geriatric syndrome, haplotypes, interleukin, polymorphisms

## Abstract

Frailty is a geriatric syndrome, characterized by a loss in functional reserve with an increase in morbidity and mortality. There are no reports that link the genetic polymorphisms between interleukin 10 *(IL10)* and frailty; for this reason, our objective was used to analyze the role of the polymorphisms of *IL10* (rs1800896, rs1800871) in the susceptibility to frailty in a Mexican population. Our study included 984 participants divided into 368 nonfrail, 309 prefrail, and 307 frail. The models for the polymorphisms rs1800896 and rs1800871 were recessive models in association with frailty (OR = 2.3, CI 95% = 1.6–3.2; OR = 1.53, CI 95% = 1.0–2.6), respectively. Two risk haplotypes were identified: ACG and CCG (*p* < .0001), and three protective haplotypes were identified: ACA, ATG, and ATA (*p* < .05). This study evaluated the relationship between *IL10* and the three subtypes of this geriatric syndrome (frail, prefrail, and nonfrail). These results support a greater susceptibility to frailty for the minor alleles of rs1800871 and rs1800896. In addition, we found two risk haplotypes supporting the participation of the *IL10* in the susceptibility for frailty in the Mexican population.

## 1 INTRODUCTION

Frailty is one of the main syndromes that affect the elderly population, impacting their quality of life, and increasing morbidity and mortality.

This syndrome is characterized by weakness, weight loss, and low physical activity, which are associated with the adverse health outcomes (Fried, Ferrucci, Darer, Williamson, & Anderson, 2004). The definition of frailty has commonality to that of aging. Both have a basis in the loss of homeostasis, though with aging the failure in homeodynamics is global, whereas with frailty the failure in homeodynamics circles around energy metabolism and neuromuscular changes (Bandeen‐Roche et al., 2006; Holliday, 1995).

Despite the growing number of studies on frailty, the physiopathological mechanisms involved in the appearance of its symptoms are not clearly known.

Inflammation has been associated as one of the physiopathological causes for the development of frailty (Chen, Mao, & Leng, 2014).

It is probable that cytokines influence frailty through various molecular pathways and cellular mechanisms that affect the synthesis of proteins, causing loss of muscle mass (Hubbard, 2009; Lang, Michel, & Zekry, 2009).

Studies have suggested that the change in cytokine expression is highly associated with frailty. An association has been reported between concentrations of cytokines such as *IL1, IL6,* and tumor necrosis factor alpha (*TNFa*) in serum with frailty in patients (Lazarus et al., 2002; Newman et al., 2001). However, other studies have not found an association between inflammation markers and susceptibility to frailty in elderly population (Yao, Li, & Leng, 2011).

Another point to consider is whether genetic differences can predispose some individuals to present frailty through inflammation.

The gene encoding *IL10* is found in chromosome 1q (1q31‐q32), and has different polymorphic sites, including three in positions ‐592A/ C (rs1800872), ‐819T/ C (rs1800871), and ‐1082A/ G (rs1800896) in the promoter region (Turner, 1997).

IL10 is an immunoregulatory cytokine, which plays a critical role in the resolution of peripheral inflammation, is produced by leukocyte cells as well as epithelial cells and endothelial cells. Therefore, some diseases may control the expression of proinflammatory cytokines, but their role in the frailty syndrome is not clear (Hutchins, Diez, & Miranda‐Saavedra, 2013; Mingomataj & Bakiri, 2016).

Cytokine levels appear to be regulated by different genetics, determined by polymorphism alleles in the genes involved in the inflammation process (Donnelly, 1999; Mohebbatikaljahi, 2009; Moore, 2001), which have already been found associated with various pathologies, such as type 1 diabetes mellitus, hypertriglyceridemia, and insulin resistance (Edwards‐Smith, 1999; Hoo, 2010; Mohebbatikaljahi, 2009). Polymorphisms of the gene in its promoter region modulate the expression of cytokines, which condition the immune response and play an important role in the evolution of the disease. It has been mentioned that in frailty, there is an important reduction in sex hormones, which play a role in the loss of body mass, bone mineral density, and muscular strength (Srinivas‐Shankar, 2010). It is also known that these hormones have a major effect on inflammatory cytokines, specifically testosterone, which increases the inflammatory cytokine Interleukin‐10 (Malkin, 2004).

In this sense, it has previously been noted that greater age also shows an increase in the cytokines, producing a chronic inflammatory process that may be responsible for the clinical symptoms of frailty.

Therefore, the present study was designed to evaluate the possible association of three polymorphisms of a single nucleotide (SNP) of *IL10* gene (‐1082 A/G, ‐819 T/C, and ‐592 A/C) with susceptibility to frailty in a well‐defined sample of elderly Mexicans, since to date there is no study in our population that has analyzed this relationship.

## 2 METHODS

### 2.1 Ethical compliance

The National Commission of Scientific Research and the IMSS Ethics Commission approved the protocol with registration number R‐2015‐785‐012.

### 2.2 Study population

The study population comes from the Study on Aging and Dementia in Mexico ("SADEM study"), a cohort study aiming to evaluate the prevalence of dementia and its risk factors among Mexican community‐dwelling elderly. The methodology of the SADEM study has been previously described (Juarez‐Cedillo et al., 2012).

Each subject included in the study was interviewed at his home by a health team previously trained to seek information on sex, age, education, marital status, activities of daily living, cognitive impairment, comorbidity, and nutritional status.

### 2.3 Definition of frailty syndrome

Frailty was defined according to Fried and Walston (Fried & Walston, 1999; Ottenbacher et al., 2005) as: (a) weight loss, defined as weight loss of over 10 pounds in the last year, (Leng, Xue, Tian, Walston, & Fried, 2007); (b) exhaustion, indicated by the affirmation of two questions “How many times in the last week did you feel this way...” “I felt everything I did was an effort”, and “I could not go on” (Reyes‐Ortega et al., 2003); (c) weakness, defined by grip strength (women 11.0 kg and men 21.0 kg); (d) slowness evaluated by walking speed recorded for 10.5 s through an 8‐foot walk; and (e) physical activity using the Spanish version of the Physical Activity Scale for the Elderly (PASE). This scale uses frequency, duration, and intensity of the previous week with a score of 0 to 793. The higher the score, the greater the physical activity (Washburn & Ficker, 1999).

### 2.4 Covariates

Cognitive function was assessed using the Mini‐Mental State Examination (MMSE) adjusted for age and education. A score of 23 or lower was used to detect cognitive impairment (Mungas, Marshall, Weldon, Haan, & Reed, 1996). Comorbidity was evaluated using the Charlson Comorbidity Index (Charlson, Pompei, Ales, & MacKenzie, 1987). Disability was assessed by a person's ability to perform Activities of Daily Living (ADL) and Instrumental Activities of Daily Living (IADL) (Katz, Ford, Moskowitz, Jackson, & Jaffe, 1963).

### 2.5 Genomic DNA

DNA extraction protocol was performed by nonenzymatic method (Lahiri & Nurnberger, 1991). The yield of the extracted DNA was quantified in spectrophotometer at 260 nm to determine sample purity. DNA material was stored at –70°C until used.

### 2.6 Gene variants of the IL10

The single‐nucleotide polymorphisms (SNPs) for this study consisted of the promoter region of the IL10 (Genbank: ID 3586) gene (rs1800896: A‐592 C; rs1800871 A‐819C and rs1800872: A‐1082G. (www.ncbi.nlm.nih.gov/SNP, BUILD135). SNPs with unknown genotype frequency and with <.05 minor allele frequency (MAF) were excluded. SNPs were genotyped using TaqMan assays on an ABI Prism 7900HT Fast Real‐time PCR system (Applied Biosystems).

### 2.7 Analysis strategies

The study population was described using mean and standard deviation (*SD*) or frequency and percentages. Student's *t* tests for continuous parameters and Chi square tests for categorical parameters were used. The Hardy–Weinberg equilibrium was evaluated for each polymorphism through Pearson's chi‐square. To test genotyping association between IL10 SNPs and frailty, logistic regression models were used to calculate the odds ratios (OR) and the 95% confidence intervals with the Bonferroni correction to compare the dominant model with the combination of two possible genotypes and three comparisons for the recessive model and the additive, as well as eight haplotype combinations for each SNP (Lewis, 2002), using the STATA 13 software for Windows® (StataCorp, 2013). The HAPLOVIEW 4.0 program (Barrett, Fry, Maller, & Daly, 2005) was used to evaluate linkage disequilibrium and its blocks. A value of *p* < .05 was considered statistically significant.

## 3 RESULTS

### 3.1 Study population

Nine hundred and eighty‐four subjects were included in the study and divided into: 368 nonfrail, 309 prefrail, and 307 frail. The clinical and demographic characteristics for each group are shown in table 1. Significant difference was observed in the age group; the frail groups classified were more likely to be older (46.9%) in comparison to the nonfrail subjects (27.4%), with *p* < .001. Women represented 57.6% of the sample, with an age of 70 ± 7.0 years. We observed significant differences between nonfrail and frail groups for the presented disabilities in the ADL and IADL scales, cognitive impairment, and the Charlson Comorbidity Index (*p* < .05).

**Table 1:** Demographic and clinical characteristic of the study groups

	Overall (*N* = 984)	Nonfrail (*n* = 368)	Prefrail (*n* = 309)	Frail (*n* = 307)	*p‐value* *
Age group, *n *(%)					
60–70 years	439 (44.6)	179 (40.8)	146 (33.3)	114 (25.9)	**<.001**
71–80 years	366 (37.2)	140 (38.3)	117 (31.9)	109 (29.8)	
≥81	179 (18.2)	49 (27.4)	46 (25.7)	84 (46.9)	
Gender, *n *(male%/female%)	417 (42.4)/567 (57.6)	139 (37.8)/229 (62.2)	148 (47.9)/161 (52.1)	130 (42.3)/177 (57.6)	**.029**
Education level, years					
0	134 (13.6)	45 (12.7)	38 (12.3)	51 (16.6)	**.008**
1–6	358 (36.4)	124 (33.8)	104 (33.7)	130 (42.3)	
7–12	286 (29.1)	106 (28.9)	101 (32.7)	79 (25.7)	
13 or more	205 (20.9)	92 (25.1)	66 (21.7)	47 (15.3)	
Marital status, *n *(%)					
Single	357 (36.3)	129 (35.1)	107 (34.6)	121 (39.4)	.285
Married	627 (63.7)	239 (64.9)	202 (65.4)	189 (60.6)	
BMI (kg/m^2^)	25.5 ± 4.2	25.4 ± 4.1	25.2 ± 4.3	24.8 ± 3.5	.172
MMSE, mean (*SD*)	26.2 ± 4.9	27.5 ± 4.7	24.4 ± 3.9	24.4 ± 4.3	**<.001**
Charlson comorbidity index					
Moderate	577 (58.6)	184 (50.0)	195 (63.1)	198 (64.5)	**<.001**
Mild	368 (37.4)	162 (44.0)	104 (33.7)	102 (33.2)	
Not severe	39 (4.0)	22 (6.0)	10 (3.2)	7 (2.3)	
Frequency of frailty components (%)					
Weight loss (%)	213 (21.6)	—	64 (6.5)	149 (15.1)	**<.001**
Exhaustion (%)	406 (41.3)		159 (16.2)	247 (25.1)	**<.001**
Weakness (%)	90 (9.1)		5 (0.5)	85 (8.6)	**.019**
Slowness (%)	285 (29.0)		79 (8.0)	206 (20.9)	**<.001**
Low physical activity (%)	266 (27.0)	—	60 (6.0)	206 (20.9)	**<.001**
Disability ≥1 IADL task (%)	273 (27.74)	45 (12.23)	69 (22.33)	159 (51.79)	**<.001**
Disability ≥1 ADL task (%)	79 (8.03)	7 (1.90)	14 (4.53)	58 (18.89)	**<.001**

Note

Data are shown as Means ± *SD* and *n* (%) number and proportion of subjects with the clinical characteristic in the groups.

* *p*‐value, were estimated using Kruskal–Wallis test and χ^2^‐test continuous variables or Pearson's test for categorical values.

The bold values indicates *p* <.05

### 3.2 Genotype frequencies of the IL10 gene

Genotype frequencies of *IL10* SNPs students in frail, prefrail, and nonfrail, are summarized in Table 2. The allelic and genotypic frequencies of the regions rs1800896, rs1800871, and rs1800872 showed differences between the group of frail and nonfrail (*p* < .001). The frequency for the genotype rs1800896 (GG) was higher for the frail group, similarly for the genotype rs1800871 (CC); we also applied a model of dominant and over‐dominant inheritance, finding significant differences when the frequencies were compared for each genotype between the frail, prefrail, and nonfrail, for the rs1800896, rs1800871 polymorphisms (dominant OR = 2.3, 95% CI = 1.6–3.2, and over‐dominant OR = 2.3, 95% CI = 1.6‐0.4) (dominant OR = 1.2 95% CI = 1.0–1.5, and over‐dominant OR = 1.3, 95% CI = 1.0–1.6), all with *p* < .000, the risk for frailty is increased only for SNP rs1800896 when adjusted by cognition, (dominant OR = 3.5, 95% CI = 2.3–5.3), and the risk is maintained when adjusted for sex, age, education, and disability level. The data were in Hardy–Weinberg equilibrium.

**Table 2:** Allele and genotype distribution of *IL10* polymorphisms and risk analysis as function of the inheritance model in frail, prefrail and nonfrail individuals

Polymorphism	Allele frequency *n *(%)		Genotype frequency *n *(%)			Model	Unadjusted OR (95% CI)	Adjusted OR (95% CI)
‐1082A/G (rs1800896)	A	G	AA	AG	GG			
Nonfrail (*n* = 368)	452 (61.4)	284 (38.6)	158 (42.9)	136 (37.0)	74 (20.1)	^a^Recessive ^b^Recessive	2.314 (1.639–3.266) 2.368 (1.646–3.407)	3.548 (2.342–5.375) 2.635 (1.781–3.898)
Prefrail (*n* = 309)	396 (64.1)	222 (35.9)	148 (47.9)	100 (32.4)	61 (19.7)			
Frail (*n* = 307)	303 (49.3)	311 (50.6)	109 (35.5)	85 (27.7)	113 (36.8)			
‐819C/T (rs1800871)	T	C	TT	TC	CC			
Nonfrail (*n* = 368)	415 (56.4)	321 (50.6)	131 (35.6)	153 (41.6)	84 (22.8)	^a^Recessive ^b^Recessive	1.538 (1.092–2.167) 1.553 (1.084–2.225)	1.480 (1.028–2.131) 1.542 (1.073–2.217)
Prefrail (*n* = 309)	356 (57.6)	262 (42.4)	117 (37.9)	122 (39.5)	70 (22.6)			
Frail (*n* = 307)	300 (48.8)	314 (51.1)	89 (29.0)	122 (39.7)	96 (31.3)			
‐592A/C (rs1800872)	A	C	AA	AC	CC			
Nonfrail (*n* = 368)	539 (73.2)	197 (26.8)	228 (62.0)	83 (22.5)	57 (15.5)	^b^Recessive	1.409 (0.948–2.092)	1.984 (1.245–3.162)
Prefrail (*n* = 309)	448 (72.5)	170 (27.5)	189 (61.2)	70 (22.6)	50 (16.2)			
Frail (*n* = 307)	421 (68.6)	193 (31.4)	177 (57.6)	67 (21.9)	63 (20.5)			

Note

a, nonfrail versus frail.

b, nonfrail versus prefrail.

OR, odd ratio; 95% CI, confidence interval *p* < .05.

The recessive model where associations were tested using logistic regression were adjusted for gender, age group (>85 years), MMES, AIDL y ADL.

In the linkage disequilibrium (LD) for polymorphisms of *IL10*, the most common haplotypes were ACG, CTG, and CCG. The LD was most detected between the rs1800872 and rs1800896 (D’: 0.88; r2: 0.008).

Table 3 shows the risk analysis by type of inheritance that proved significant for each frailty domain, showing a significant risk for the domain of weight loss, weakness, and slowness in the recessive model of rs1800896, while for the recessive model of rs1800871 only observed a significant risk to exhaustion.

**Table 3:** Risk analysis as function of the inheritance mode by each frailty domain

Frailty components (%)	‐1082A/G (rs1800896); Recessive Model		‐819C/T (rs1800871) Recessive Model	
	OR (95% CI)	*p*	OR (95% CI)	*p*
Weight loss (%)	2.043 (1.473–2.832)	<.001	1.391 (0.995–1.945)	.054
Exhaustion (%)			1.354 (1.014–1.809)	.040
Weakness(%)	1.828 (1.160–2.882)	.009		
Slowness (%)	1.828 (1.348–2.478)	<.001		

Note

Only significant risks are shown, *p* < .05.

### 3.3 Haplotype association test

In the association tests we identified eight haplotypes, of which two associated significantly with frailty risk: ACG (OR = 3.02, 95% CI: 2.08–4.437), CCG (OR = 1.88, 95% CI = 1.18–3.00), *p* < .005, as presented in Table 4. The haplotypes ACG, CTG, and CCG were protective, but not significantly for susceptibility of frailty syndrome, *p* < .05.

**Table 4:** *IL10* haplotype frequencies in frail and nonfrail

Haplotypes	Block	Frail (*n* = 307)	Nonfrail (*n* = 368)	OR (95% CI)	*p*‐value
H1	ACA	0.201	0.257	0.725 (0.560–0.938)	.014
H2	ATG	0.164	0.209	0.744 (0.564–0.982)	.036
H3	ATA	0.154	0.205	0.718 (0.542–0.952)	.021
H4	A**CG**	**0.164**	**0.061**	**3.023 (2.089–4.374)**	**<.001**
H5	CTG	0.101	0.074	1.418 (0.968–2.078)	.071
H6	CCA	0.070	0.075	0.931 (0.615–1.408)	.734
H7	CTA	0.067	0.076	0.869 (0.572–1.320)	.509
H8	C**CG**	**0.077**	**0.042**	**1.885 (1.182–3.006)**	**.007**

Note

The order of the polymorphisms in the haplotypes is according to the positions in the chromosome (rs1800872, rs1800871 and rs1800896).

Construct eight haplotypes.

Bold numbers indicate significant associations.

Abbreviations: 95% CI, confidential interval; OR, odds ratio.

## 4 DISCUSSION

Inflammatory processes have been implicated as responsible for the physiopathological changes that cause the symptoms of frailty (Lang et al., 2009; Yao et al., 2011). The origin of inflammation is the activation of immune cells, among which is interleukin‐10 (IL‐10), whose role is to reduce and regulate cellular inflammatory response, so it is expected to play an important role in susceptibility to frailty.

We studied the relationship between genetic variants of the IL‐10 gene and susceptibility to frailty by analyzing three polymorphic sites located in the promoter region (Turner, 1997). Our results show a significant association for polymorphism rs1800896 with frailty in the recessive model that is maintained after adjusting for variables such as age, sex, and comorbidities.

These findings reinforce the participation of inflammation in the development of symptoms such as loss of muscular strength, as a consequence of a loss of muscle mass among frail patients (Lang et al., 2009; Yao et al., 2011).

Allele A was overexpressed more frequently for polymorphism rs1800896 in study participants with frailty compared with prefrail and controls, so the presence of allele A increases susceptibility to frailty. Likewise, minor allele C for rs1800871 was presented less frequently in controls and is also associated with an increased risk of frailty.

With our results, we offer more information to clarify the epigenetic aspects of the development of frailty among elderly Mexicans.

The frequencies of the genotypes for *IL10* show a behavior similar to that reported for the general population in Mexico (Vargas‐Alarcon et al., 2012) in relation to the polymorphisms analyzed (rs1800872, rs1800871, rs1800896). There are no reports to date which indicate their association with the risk of frailty in Mexican or other populations.

We found a highly significant relation between these polymorphisms and risk of frailty, as well as with haplotypes ACG and CCG, which are associated with high and low levels of *IL10* production (Scarpelli et al., 2006; Suárez, Castro, Alonso, Mozo, & Gutiérrez, 2003), which may explain the effect of inflammation on the loss of muscle mass, relevant clinical manifestation in the frailty syndrome (Wang & Casolaro, 2014).

Haplotype association test showed a statistically significant reduction in risk with haplotypes ACA, ATG, ATA, while haplotypes ACG and CCG suggested an association with the risk of developing frailty. These findings may be important, given the genetic diversity of populations. (Dixon, Marazita, Beaty, & Murray, 2011).

The results shed important light, given that this is the first study to indicate the association of this gene with various phenotypes of frailty, considering an adequate sample size to sustain the findings.

It has been noted that the frailty syndrome is a complex entity, where the participation of environmental and genetic factors play an important role in its development and evolution (De Martinis, Franceschi, Monti, & Ginaldi, 2006; Leng et al., 2007; Leng, 2004). In our study, the additive effect of the risk haplotypes was determined as ACG and CCG for frailty, noting that the genetic variability depends on the phenotype for frailty more than age, and risk was maintained after adjustment for the aforementioned environmental factors. This suggests that if inflammation is a characteristic condition for aging, it may be closely related with susceptibility to frailty, and may be a marker of this syndrome.

The strength of this study is that it is the first to consider analyzing the effect of inflammation mediated by *IL10* and the phenotypes for frailty (frail, prefrail, and controls), showing the importance of the development of strategies directed towards modulating inflammatory response in order to limit the prevalence of frailty syndrome among the elderly.

## 5 CONCLUSION

These results support a greater susceptibility to frailty for the minor alleles of the rs1800871 and rs1800896. In addition, we found two risk haplotypes, supporting the participation of the IL‐10 in the susceptibility for frailty in the Mexican population. These findings should be strengthened with the study of other markers.

## CONFLICT OF INTEREST

The authors declare no conflict of interest.

## AUTHOR CONTRIBUTIONS

TJC was involved in design and writing of the manuscript. GVA was involved in analysis of data. NMR was involved inthe interpretation of data and literature search. JMF was involved in analysis of data. JCE was involved in data collection and genetic analysis. JEP was involved interpetation of results.
